# An Early Fire Detection Algorithm Using IP Cameras

**DOI:** 10.3390/s120505670

**Published:** 2012-05-03

**Authors:** Leonardo Millan-Garcia, Gabriel Sanchez-Perez, Mariko Nakano, Karina Toscano-Medina, Hector Perez-Meana, Luis Rojas-Cardenas

**Affiliations:** 1 Graduate School, ESIME-Culhuacan, National Polytechnic Institute, Av. Santa Ana no. 1000, Col. San Francisco Culhuacan, Mexico D.F., 04430, Mexico; E-Mails: millan.galeis@gmail.com (L.M.-G.); mnakano@ipn.mx (M.N.); ltoscano@ipn.mx (K.T.-M.); hmperezm@ipn.mx (H.P.-M.); 2 Electric Engineering Department, Metropolitan Autonomous University, Iztapalapa Campus, Mexico D.F., 09340, Mexico; E-Mail: lmrc@xanum.uam.mx

**Keywords:** early fire detection, smoke detection, DCT, DCT inter-transformation, video surveillance, IP camera

## Abstract

The presence of smoke is the first symptom of fire; therefore to achieve early fire detection, accurate and quick estimation of the presence of smoke is very important. In this paper we propose an algorithm to detect the presence of smoke using video sequences captured by Internet Protocol (IP) cameras, in which important features of smoke, such as color, motion and growth properties are employed. For an efficient smoke detection in the IP camera platform, a detection algorithm must operate directly in the Discrete Cosine Transform (DCT) domain to reduce computational cost, avoiding a complete decoding process required for algorithms that operate in spatial domain. In the proposed algorithm the DCT Inter-transformation technique is used to increase the detection accuracy without inverse DCT operation. In the proposed scheme, firstly the candidate smoke regions are estimated using motion and color smoke properties; next using morphological operations the noise is reduced. Finally the growth properties of the candidate smoke regions are furthermore analyzed through time using the connected component labeling technique. Evaluation results show that a feasible smoke detection method with false negative and false positive error rates approximately equal to 4% and 2%, respectively, is obtained.

## 1. Introduction

Early fire detection can help to alert of and prevent disasters that generate great economic damages and human losses. The combustion of objects usually begins with the emission of smoke, even before catching fire; therefore the presence of smoke is an essential factor for early fire detection. The features that describe the smoke depend on chemical properties of the combusting object, the fire temperature, the amount of oxygen, and so on. Generally the smoke color range goes from white to white-bluish when the combustion temperature is low, and from gray to black when the temperature rises to ignition. The most common smoke detectors are based on infrared or ultraviolet cameras, while other detection techniques are based on the analysis of particles, temperature, relative humidity and air transparency. Those systems are activated until the smoke particles or flames are very close to the fire detector device, moreover those devices cannot provide more information regarding to the exact location of fire, magnitude, growth rate and so on [1]. To provide more accurate and reliable smoke detection, some video processing-based detection systems have been proposed.

Generally the video processing-based fire detection algorithms are carried out using two principal characteristics of fire, which are flame and smoke. Almost all fire detection algorithms in the literature perform a pixel level analysis using some flame and/or smoke properties, such as the flame/smoke color, flickering nature, loss of background edges in frames, among others. In [2], authors proposed a method for fire detection using a multilayer neural network (MNN) with a back-propagation algorithm, which is trained using the color property of flames presented in the HSI (Hue-Saturation-Intensity) color space. This algorithm analyses the color of each pixel to determine if some pixels present the flame features or not. In [3] and [4], the Hidden Markov Models (HMM) and the discrete wavelet transform (DWT) are used to detect flickering pixels that indicate the presence of flames. Generally the presence of flames may indicate more a serious fire situation than the presence of smoke only. Therefore for early fire detection purposes, smoke detection schemes may be more efficient.

In [5] and [6], the authors use of a method for detecting smoke based on the loss of high frequencies using HMM and DWT. In [1] the RGB image sequences are analyzed to detect smoke using its chromaticity and grade of disorder. The proposal of [7] combines several dynamic and static smoke features, such as growth, disorder, flicking frequency and the energy of wavelet transform, and then this combined information is used to train a MNN to detect the presence of smoke. In [8], a smoke detection algorithm analyses the smoke candidate area using the smoke motion direction in a cumulative manner through the video sequences. The algorithm in [9] seeks to detect the smoke and the flame inside a tunnel, in which the fire detection is based on the extracted motion area using a background image and the motion history of images, as well as the invariant moments. The main problem of this application is the large amount of movement generated by cars and heavy air currents. In the smoke detection algorithm proposed by [10], the smoke is considered as a type of texture pattern, which is extracted using local binary patterns (LBP) that are commonly used as texture classifier. These LBP are then used to train a MNN which determines the presence of smoke. In [11], using the smoke color property defined in [1] and smoke motion detected by optical flow algorithm, a MNN is trained to detect the presence of smoke. It is worth noting that all fire detection algorithms mentioned above operate in the spatial domain, analyzing pixel values of each frame of video.

Recently the use of IP cameras in video surveillance has grown significantly, because video surveillance systems based on IP technology are easy to implement at low cost due to the use of cabling and wireless Internet infrastructure already present in many companies [12]. Moreover, an IP camera not only captures sequences of images, but also has its own processor, memory and operating system, allowing loaded programs to process the captured information without the need of additional computer equipment. IP cameras can also be connected to form networks, making a video surveillance system more reliable. Generally the information provided by IP camera is encoded data in several formats, such as Motion-JPEG (MJPEG), H.264, *etc.* [12].

The use of IP technology for fire detection offers several advantages, for example IP-camera networks can detect fire origin, magnitude and propagation in more accurate manner compared with a single video surveillance system. However to efficiently use the IP technology for fire detection purposes, the smoke detection algorithm must perform directly in the Discrete Cosine Transform (DCT) domain, because decoding (from DCT domain to spatial domain) and possible encoding (from spatial domain to DCT domain) are considerably high time consuming processes. However almost all fire detection algorithms including those proposed in [1–11] are carried out in the spatial domain, analyzing the value of each pixel or block of pixels. Therefore any implementation of these algorithms in IP technology requires considerably high extra processing time.

This paper proposes a smoke detection algorithm, which is an extended version of that presented in UCAmI'11 [13]. The proposed algorithm operates directly in DCT domain and can be implemented in IP camera-based surveillance system. The proposed algorithm detects the presence of smoke using several smoke features, such as color, motion and spreading characteristics, which are extracted directly from DCT coefficients to avoid the decoding process. To increase the resolution of video frames without significantly increasing the computational cost, fast inter-transformation of DCT coefficients proposed in [14] and [15] are used. The computer simulation results show the efficiency and high smoke detection rates of the proposed algorithm. The rest of this paper is organized as follows: Section 2 describes the proposed video processing-based smoke detection scheme. The experimental results and discussions are shown in Section 3, following by conclusions in Section 4.

## 2. Proposed Video Processing-Based Smoke Detection Scheme

The proposed smoke detection scheme is designed to work efficiently in an IP camera-based system, in which the sequence encoded by the MJPEG codec is available as input data for the smoke detection algorithm. Recently, IP cameras with H.264 codec have been developed; however the cost of those IP cameras is much higher than that of IP cameras with MJPEG codec and we consider that the high compression rate offered by H.264 is not necessary for the smoke detection tasks, because it is not necessary to store and/or transmit the captured video sequences between the IP camera modules and the main computer systems. Also MJPEG codec offers higher quality of frames than H.264 codec. Therefore we decided that an MJPEG based IP camera module is the most adequate platform for efficient smoke detection scheme considering computational and economical cost, as well as the frame quality. Although the proposed scheme is designed for MJPEG codec system, it can be adapted to H.264 with minor modifications.

**Figure 1.**
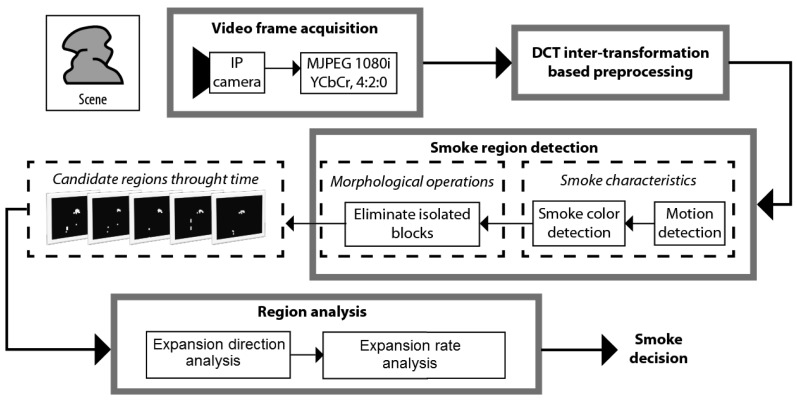
Block diagram of the proposed smoke detection scheme.

The block diagram of the proposed smoke detection scheme is shown in Figure 1, which is composed of four stages: video frames acquisition stage, DCT inter-transformation based preprocessing stage, smoke region detection stage and region analysis stage. In the video frames acquisition stage, each frame of size 1,920 × 1,080 pixels is captured by an IP camera and encoded using an standard JPEG codec, in which bi-dimensional DCT is applied to non-overlapped blocks of 8 × 8 pixels of each frame. In the preprocessing stage, the DCT inter-transformation is applied to all DCT blocks of 8 × 8 coefficients of each frame to get DCT blocks of 4 × 4 coefficients without using the inverse DCT (IDCT). In the smoke region detection stage, using the DC values of each DCT block of the 4 × 4 coefficients of several consecutive frames, motion and color properties of smoke are analyzed to get the smoke region candidates. The candidate regions are processed using morphological operations to eliminate isolated blocks. Using the connected component labeling algorithm, the smoke expansion properties of the candidate regions are analyzed through time to discard non-smoke regions. All stages, except the video frames acquisition stage, are described in following subsections.

### 2.1. DCT Inter-Transformation Based Preprocessing

As mentioned before, an IP camera module provides DCT blocks of 8 × 8 coefficients of each frame; however this block size is too large for accurate analysis of smoke features and it is necessary to use a smaller block size. Traditionally if a DCT block with a size different from a current block size is required, the IDCT must be computed and then a new DCT with the required block size is re-calculated. These processes are highly time consuming operations. In [14,15], inter-transformation of DCT coefficients is proposed, in which the relationship between DCT coefficients of different block sizes is established.

Consider the DCT coefficients of a block *B* of size S × S, which are given by:

(1) $$\begin{array}{c} C\left(u,v\right)=\sqrt{\frac{2}{S}}\alpha (v)\sum_{q=0}^{S-1}\left(\sqrt{\frac{2}{S}}\alpha (u)\sum_{p=0}^{S-1}B\left(p,q\right)\cos\left(\frac{(2p+1)u\pi }{2S}\right)\right)\cos\left(\frac{(2q+1)v\pi }{2S}\right)\\ =\frac{2}{S}\alpha (v)\alpha (u)\sum_{q=0}^{S-1}\left(\sum_{p=0}^{S-1}B\left(p,q\right)\cos\left(\frac{(2p+1)u\pi }{2S}\right)\right)\cos\left(\frac{(2q+1)v\pi }{2S}\right) \end{array}$$

where

$$u,v=1,2,\ldots S,\alpha (u)\alpha (v)=\{\begin{array}{cc} \frac{1}{\sqrt{2}} & u=0\;\text{o}\;v=0\\ 1 & \text{otherwise} \end{array}$$

and defining a matrix *M_S_* of S × S as:

(2) $$\begin{array}{l} M_{S}\left(h,l\right)=\{\begin{array}{cc} \frac{1}{\sqrt{S}} & h=0\\ \sqrt{\frac{2}{S}}\cos\frac{\pi (2l+1)h}{2S} & h\neq 0 \end{array}\\ h,l=0,1,\cdots S-1 \end{array}$$

It follows that:

(3) $$M_{S}=\sqrt{\frac{2}{S}}\left[\begin{array}{ccccc} \frac{1}{\sqrt{2}} & \frac{1}{\sqrt{2}} & \frac{1}{\sqrt{2}} & \cdots & \frac{1}{\sqrt{2}}\\ \cos\frac{\pi }{2S} & \cos\frac{3\pi }{2S} & \cos\frac{5\pi }{2S} & \cdots & \cos\frac{(2S-1)\pi }{2S}\\ \cos\frac{2\pi }{2S} & \cos\frac{6\pi }{2S} & \cos\frac{10\pi }{2S} & \cdots & \cos\frac{(2S-1)2\pi }{2S}\\ \vdots & \vdots & \vdots & \vdots & \vdots \\ \cos\frac{(S-1)\pi }{2S} & \cos\frac{3(S-1)\pi }{2S} & \cos\frac{5(S-1)\pi }{2S} & \cdots & \cos\frac{(2S-1)(S-1)\pi }{2S} \end{array}\right]$$

Using Equation (3), we can rewrite Equation (1) as:

(4) $$C=M_{S}\times B\times {M_{S}}^{T}$$

Multiplying Equation (4) on the left by $M_{S}^{-1}$ and on the right by ${\left(M_{S}^{T}\right)}^{-1}$, we get:

(5) $$B={M_{S}}^{-1}\times C\times {\left(M_{S}^{T}\right)}^{-1}$$

Considering that the block *B* is divided in 4 sub-blocks of size *S*/2 × *S*/2 and denoting each sub-block by *_S_B_qr_*, *q*,*r* = 1,2, we can get $B=\left[\begin{array}{cc} sB_{11} & sB_{12}\\ sB_{21} & sB_{22} \end{array}\right]$.

The coefficients of DCT of each sub-block and its inverse transform can be expressed in the same manner as Equations (4) and (5):

(6) $$C_{qr}=M_{\frac{S}{2}}\times sB_{qr}\times {M_{\frac{S}{2}}}^{T}$$

(7) $$sB_{qr}={\left(M_{\frac{S}{2}}\right)}^{-1}\times C_{qr}\times {\left({M_{\frac{S}{2}}}^{T}\right)}^{-1}$$

Substituting Equation (7) in Equation (5), we get:

(8) $$\begin{array}{l} B={M_{S}}^{-1}\times C\times {\left(M_{S}^{T}\right)}^{-1}=\left[\begin{array}{ll} {\left(M_{\frac{S}{2}}\right)}^{-1}\times C_{11}\times {\left({M_{\frac{S}{2}}}^{T}\right)}^{-1} & {\left(M_{\frac{S}{2}}\right)}^{-1}\times C_{12}\times {\left({M_{\frac{S}{2}}}^{T}\right)}^{-1}\\ {\left(M_{\frac{S}{2}}\right)}^{-1}\times C_{21}\times {\left({M_{\frac{S}{2}}}^{T}\right)}^{-1} & {\left(M_{\frac{S}{2}}\right)}^{-1}\times C_{22}\times {\left({M_{\frac{S}{2}}}^{T}\right)}^{-1} \end{array}\right]\\ ={\left[\begin{array}{ll} M_{\frac{S}{2}} & 0\\ 0 & M_{\frac{S}{2}} \end{array}\right]}^{-1}\left[\begin{array}{ll} C_{11} & C_{12}\\ C_{21} & C_{22} \end{array}\right]{\left[\begin{array}{ll} M_{\frac{S}{2}}^{T} & 0\\ 0 & M_{\frac{S}{2}}^{T} \end{array}\right]}^{-1} \end{array}$$

And substituting Equation (8) in Equation (4), it follows:

(9) $$C=M_{S}\times {\left[\begin{array}{cc} M_{\frac{S}{2}} & 0\\ 0 & M_{\frac{S}{2}} \end{array}\right]}^{-1}\times \left[\begin{array}{ll} C_{11} & C_{12}\\ C_{21} & C_{22} \end{array}\right]\times {\left[\begin{array}{cc} M_{\frac{S}{2}}^{T} & 0\\ 0 & M_{\frac{S}{2}}^{T} \end{array}\right]}^{-1}\times M_{S}^{T}$$

To simplify we introduce a matrix *A* of *S* × *S* given by:

(10) $$A=\left[\begin{array}{cc} M_{\overset{S}{\;}/\underset{2}{\;}} & 0\\ 0 & M_{\overset{S}{\;}/\underset{2}{\;}} \end{array}\right]\times {M_{S}}^{-1}$$

Considering *A^T^* = *A*^−^*^1^*, the relationship between DCT coefficients of a block of *S* × *S* and those of its sub-blocks of *S/2* × *S/2* is obtained as:

(11) $$\left[\begin{array}{ll} C_{11} & C_{12}\\ C_{21} & C_{22} \end{array}\right]=A\times C\times A^{-1}$$

(12) $$C=A^{-1}\times \left[\begin{array}{ll} C_{11} & C_{12}\\ C_{21} & C_{22} \end{array}\right]\times A$$

According to [14], this DCT inter-transformation is four times faster than the traditional IDCT-DCT operation. In the proposed smoke detection scheme, firstly the DCT inter-transformation is applied to each frame to get *S/2* × *S/2* DCT blocks directly from *S* × *S* DCT blocks, where *S* = 8 because an IP camera is used.

### 2.2. Smoke Region Detection Stage

In the smoke region detection stage, some smoke block candidates are estimated using the motion and color properties of smoke. This stage receives DCT blocks of *S_b_* × *S_b_* coefficients previously calculated by the preprocessing stage of each frame, which is composed of three channels: luminance channel (*Y*) and two chrominance channels (*C_b_* and *C_r_*). The motion property of smoke is analyzed using only the luminance channel *Y*, and the smoke color property is analyzed using two chrominance channels *C_b_* and *C_r_*.

#### 2.2.1. Smoke Motion Analysis

Considering that the DC coefficient of DCT block of *S_b_* × *S_b_* is *S_b_* times the average value of the block in spatial domain as shown by Equation (13), only the DC value of each DCT block is considered for motion analysis:

(13) $$\begin{array}{l} C(0,0)=\sqrt{\frac{2}{S_{b}}}\alpha (0)\sum_{q=0}^{S_{b}-1}\left(\sqrt{\frac{2}{S_{b}}}\alpha (0)\sum_{p=0}^{S_{b}-1}B(p,q)\cos\left(\frac{(2p+1)\times 0\times \pi }{2S_{b}}\right)\right)\cos\left(\frac{(2p+1)\times 0\times \pi }{2S_{b}}\right)\\ \;=S_{b}\times \left(\frac{1}{S_{b}^{2}}\sum_{q=0}^{S_{b}-1}\sum_{p=0}^{S_{b}-1}B(p,q)\right) \end{array}$$

Considering that $Y_{t}^{DC}(x,y)$ is the DC value of (*x*,*y*)-th block of the luminance channel *Y* in frame *t*, each DCT block is classified into motion or static blocks using blocks $Y_{t-1}^{DC}(x,y)$ and $Y_{t}^{DC}(x,y)$. This classification is given by:

(14) $$f_{m}\left(Y_{t-1}^{DC}(x,y),Y_{t}^{DC}(x,y)\right)=\{\begin{array}{rr} 1 & \text{if}\;th_{1}<\frac{1}{S_{b}}\left|Y_{t-1}^{DC}(x,y)-Y_{t}^{DC}(x,y)\right|<th_{2}\\ 0 & \text{otherwise} \end{array}$$

where the matrix *f_m_* is a binary matrix of size *M* × *N* (*M* = 1,920/*S_b_* and *N* = 1,080/*S_b_*), indicating a moving smoke block with value ‘1’, otherwise ‘0’, and *th_1_* and *th_2_* are two threshold values considering the general motion speed presented by smoke. The smoke motion and any other moving objects produce changes in the luminance value of a given block in two consecutive frames. Then, to avoid a wrong detection, taking into account the smoke movement speed, we set a suitable range given by the threshold *th_1_* and *th_2_*, whose values are experimentally determined as 12 and 80, respectively.

Another important result obtained from Equation (14) is the fact that, the proposed smoke motion analysis is robust to different scene illumination produced by various weather conditions, because it depends only on the difference of luminance of two consecutive frames; then from Figure 1 it follows that the proposed smoke detection algorithm is also robust to scene illumination changes.

#### 2.2.2. Smoke Color Analysis

The color is another important feature of smoke; therefore this feature has been used commonly in several smoke detection algorithms [8,11]. Almost all algorithms used Chen's smoke color model [1], in which the smoke color is determined using RGB color space-based rules. The first rule is based on the fact that the smoke color is gray, which means intensities of three color-channels are approximately the same. The second rule determines that the gray intensity must be between 80 and 220. This range indicates that the smoke color is neither so white nor so black. These rules, proposed by [1], are given by Equation (15):

(15) $$\begin{array}{l} \text{Rule}\;1:R\pm \alpha =G\pm \alpha =B\pm \alpha \\ \text{Rule}\;2:80\leq \frac{R+G+B}{3}\leq 220 \end{array}$$

where 15 ≤ *α* ≤ 20. If both rules are satisfied, then the pixel is considered as smoke.

In the proposed algorithm, which is performed in MJPEG domain by the IP camera, the available color space is YC_b_C_r_ instead of RGB color space. Therefore the smoke color model proposed by Chen *et al.* [1] must be adapted as follows:

(16) $$\begin{array}{c} \text{Rule}1:{\left(C_{b}^{DC}(x,y)-128\right)}^{2}+{\left(C_{r}^{DC}(x,y)-128\right)}^{2}\leq {\hat{\alpha }}^{2}\\ \text{Rule}2:Th_{3}\leq Y^{DC}(x,y)\leq Th_{4} \end{array}$$

where $C_{b}^{DC}(x,y)$, $C_{r}^{DC}(x,y)$, and *Y^DC^* (*x*, *y*) are the DC values of two chrominance and luminance channels of (*x*,*y*)-th block, and applying the linear transform between RGB and YC_b_C_r_, it follows that *α̂* = 10, *Th_3_* = 80 and *Th_4_* = 220. Using the same manner of Chen's model, if both rules are satisfied, then the (x,y)-th block is considered as smoke block by color property, that is $f_{c}=(Y^{DC}(x,y),C_{b}^{DC}(x,y),C_{r}^{DC}(x,y))=1$, otherwise $f_{c}=(Y^{DC}(x,y),C_{b}^{DC}(x,y),C_{r}^{DC}(x,y))=0$.

**Figure 2.**
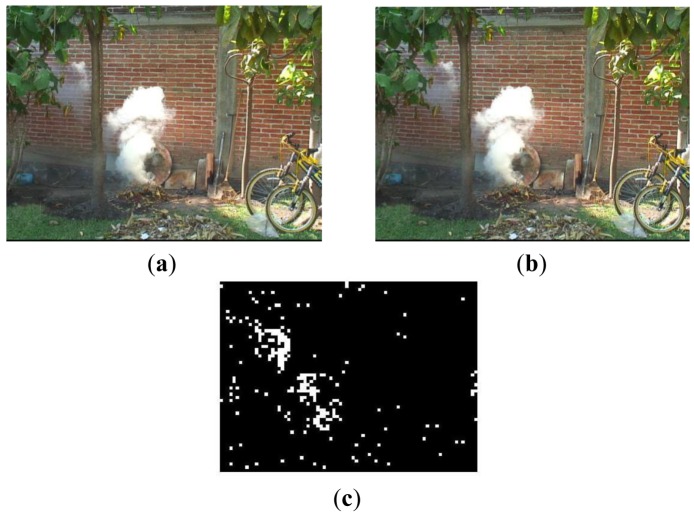
Detection of candidate smoke blocks; (**a**) and (**b**) consecutive frames and (**c**) binary image *B_t_*.

Once both smoke feature analyses are concluded, the blocks that satisfy both smoke features are considered as smoke candidate regions, as follows:

(17) $$B_{t}(x,y)=f_{m}\left(Y_{t-1}^{DC}(x,y),Y_{t}^{DC}(x,y)\right)\wedge f_{c}(Y^{DC}(x,y),C_{b}^{DC}(x,y),C_{r}^{DC}(x,y))$$

where operator ‘⋀’ denotes logical-and. If *B_t_*(*x*, *y*) is ‘1’ then (*x,y*)-th block is smoke candidate block and moreover is analyzed in next stage, otherwise the block is discarded. Figure 2 shows two consecutive frames, and binary image calculated by Equation (17), in which the candidate smoke blocks are represented by white block.

#### 2.2.3. Elimination of Isolated Blocks

**Figure 3.**
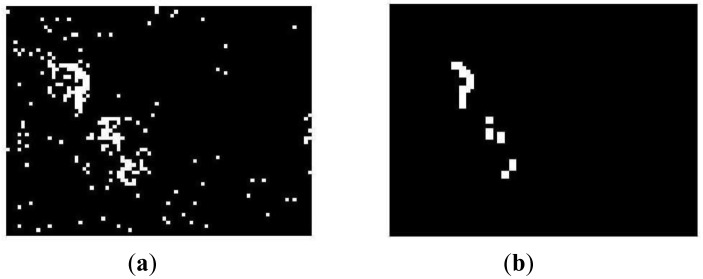
Noise reduction by morphological opening operation. (**a**) binary image obtained by Equation (17) and (**b**) binary image after morphological opening.

Illumination variations and motion caused by wind are the principal factors of erroneous block detection; however these erroneous blocks can be detected easily because these blocks are generally isolated. Taking in account the expansion property of smoke, which occupies several connected blocks, the isolated blocks can be considered as erroneous blocks. To eliminate the isolated blocks, the morphological opening operation based on the dilation and erosion is applied to the binary matrix *B_t_* obtained by Equation (17), which is given by:

(18) $$M_{t}=B_{t}\circ W=(B_{t}⊗W)⊕W$$

where ○, ⊗ and ⊕ are opening, erosion and dilation operators, respectively, and *W* is 2 × 2 square structuring element. Figure 3 shows a noisy binary image *B_t_* and a binary image *M_t_* is the result of the morphological opening operation given by Equation (18). We can observe that many isolated blocks are eliminated efficiently.

### 2.3. Region Analysis Stage

Once the smoke candidate regions are detected, the behavior of these regions through the several frames must be analyzed, because some objects possess similar properties to smoke. After the binary images *M_t_(t = 0,…T)* from each consecutive frame are obtained by Equation (18), the smoke candidate regions composed of several connected blocks are detected and labeled using connected component labeling algorithm with connectivity-4. Each candidate region is denoted by $A_{t}^{k}$, *k* = 1,2,…*K*, where *k* means the label number and *K* is a total number of candidate regions in the binary image *M_t_* of time *t*. Considering that smoke has a property of continuously expansion, the corresponding smoke regions $A_{t-1}^{k}$ and $A_{t}^{k}$ of the consecutive binary images *M_t-1_* and *M_t_*, respectively, present an expansion with overlapping. This property can efficiently discriminate smoke from other moving object with similar color, such as car light, moving person with gray clothes, *etc.* To analyze this smoke property, each region is updated using Equation (19):

(19) $$A_{t}^{k}=\{\begin{array}{cc} A_{t-1}^{k}\cup A_{t}^{k}, & \text{if}\;A_{t-1}^{k}\cap A_{t}^{k}\neq \phi \\ A_{t}^{k}, & \text{otherwise} \end{array}k=1,2,\ldots ,K$$

where *ϕ* denotes null set, while ‘∪’ and ‘∩’ are union and intersection operators, respectively. Thus the new binary image *M̂_t_* becomes:

(20) $${\hat{M}}_{t}=\overset{K}{\underset{k=1}{\cup }}A_{t}^{k}$$

**Figure 4.**
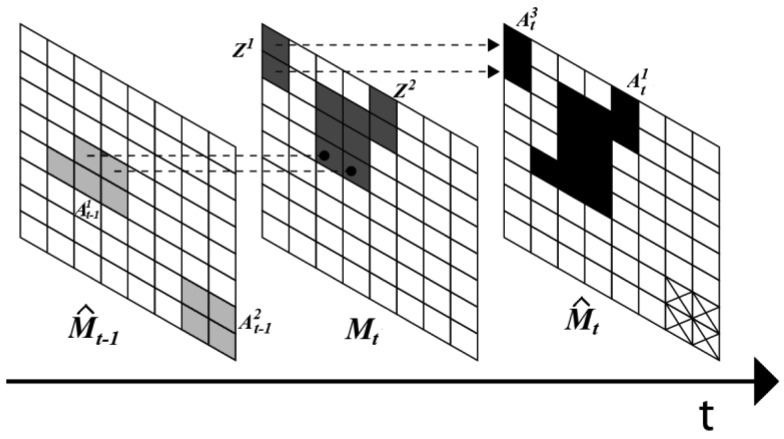
Binary images *M̂_t_*_−1_ and *M̂_t_* obtained applying Equation (19) to each candidate region.

Figure 4 illustrates this update operation of each smoke candidate region.

In Figure 4, there are two regions ${\text{A}}_{\text{t}-1}^{1}$ and ${\text{A}}_{\text{t}-1}^{2}$ in *M̂_t_*_−1_. In *M_t_*, two candidate regions *Z^1^* and *Z^2^* are extracted using the smoke properties mentioned above. According to Equation (19), the region of ${\text{A}}_{\text{t}-1}^{1}$ is expanded by the union operation with *Z^2^* due to ${\text{A}}_{\text{t}-1}^{1}\cap Z^{2}\neq \phi$, while the region ${\text{A}}_{\text{t}-1}^{2}$ disappears because it is not intersected with any candidate region in *M_t_*. The new candidate region *Z^1^* is registered as new region ${\text{A}}_{\text{t}}^{3}$ in the binary image *M̂_t_*.

**Figure 5.**
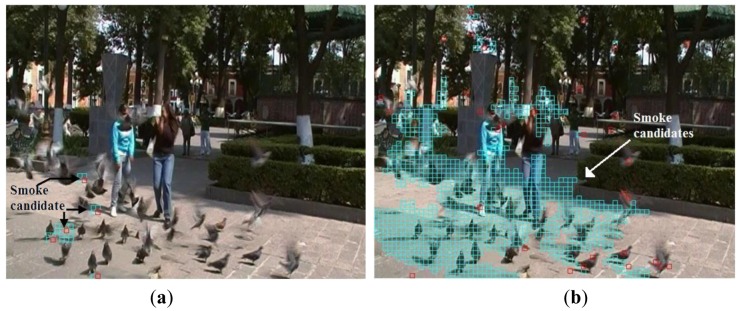
Smoke candidate region detection. (**a**) using morphological operation (**b**) without morphological operation.

To achieve an accurate detection of smoke candidate regions, it is essential to use morphological operations to eliminate noise in the frame under analysis. This is because without noise elimination, small noisy regions may be overlapped through the time producing wrong smoke candidate regions, degrading the system performance. Figure 5 shows an example of smoke candidate region detection with and without morphological operations in absence of smoke, indicating clearly the importance of using the morphological operations.

**Figure 6.**
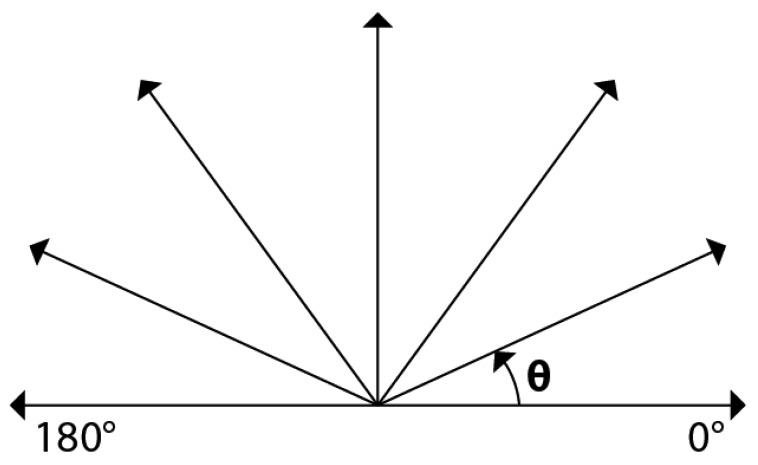
Expansion direction of smoke regions.

Considering that generally smoke expands upwards, the expansion direction of each candidate region is estimated, and using this direction, some regions with different expansion directions can be discarded. In this process firstly the centroids of each candidate region of two consecutive time intervals are calculated. Next the motion vector is estimated as the difference between both centroids. Then if the motion vector angle θ is larger than 0° and smaller than 180°, as shown by Figure 6, the region under analysis can be considered as potential smoke.

After discarding the non-smoke regions that do not satisfy the expansion direction criterion, the expansion area is analyzed, as follows:

(21) $$R^{k}(t)=\text{number}\left((x,y)|\left(x,y)\in A_{t}^{k}(x,y\right)\right),k=1,2,\ldots ,K$$

where *number*(*y*) denotes the number of element of *y*. Next, if *R^k^*(*t*) is an increasing function during a given time interval, *th_5_*, and the condition given by Equation (22) is satisfied then ${\text{A}}_{\text{t}}^{\text{k}}(x,y)$ is considered as a smoke region.

(22) $$R^{k}(t+th_{5})\geq th_{6}\times R^{k}(t)$$

where *th_5_* is monitoring interval which, for early smoke detection, is set to 1 second and *th_6_* is growing rate of the smoke region which was set to 1.5.

**Figure 7.**
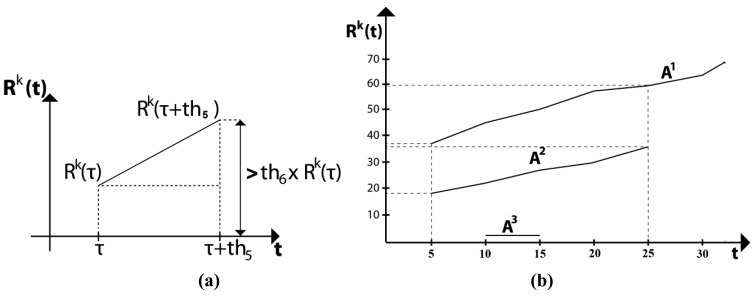
(**a**) Description of the condition given by Equation (22) and (**b**) An example of the expansion of three candidate smoke regions *A^1^*, *A^2^* and *A^3^*.

Figure 7(a) illustrates the condition of smoke region given by Equation (22) and Figure 7(b) shows an example of three candidate smoke regions. From this figure it follows that the areas of *A^1^* and *A^2^* grow through time, for example *A^1^* grows from 37 to 60 blocks in 20 frames, while *A^2^* grows from 19 to 36 blocks during this time. Considering that the frame rate is 20 frames/sec, it follows that *th_5_* is equal to 20, and these two regions satisfy the condition in Equation (22), because 60 ≥ 37 × 1.5 = 55.5 and 36 ≥ 19 × 1.5 = 28.5. And then these regions can be considered as smoke region. On the other hand, *A^3^* remains almost constant during five frames and later this region disappears, therefore it is not considered a smoke region.

## 3. Results and Discussion

**Figure 8.**
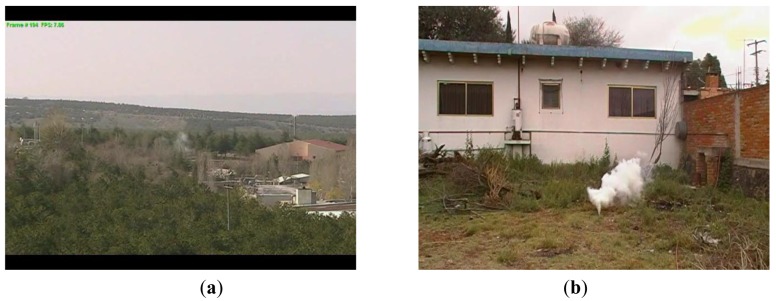

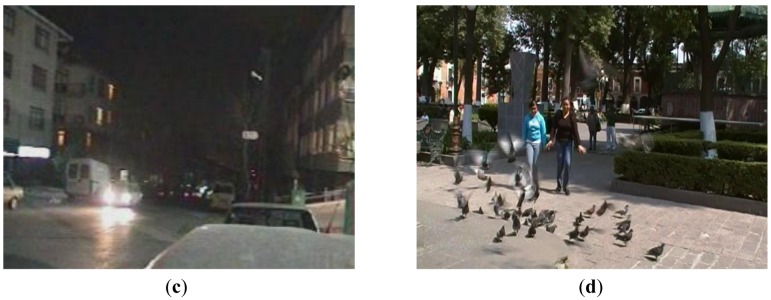
Some of video sequences used in evaluation of the proposed algorithm.

The performance of the proposed algorithm is evaluated using 50 videos with smoke and other 50 videos without smoke which are available in [16,17]. Figure 8 shows some frames of video sequences used in evaluation, in which (a) and (b) are video frames with smoke and (c) and (d) are video frames without smoke.

**Figure 9.**
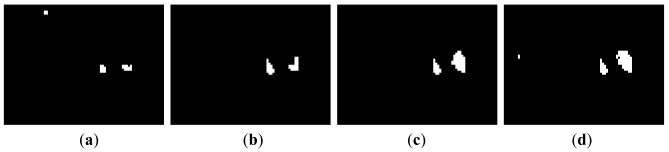

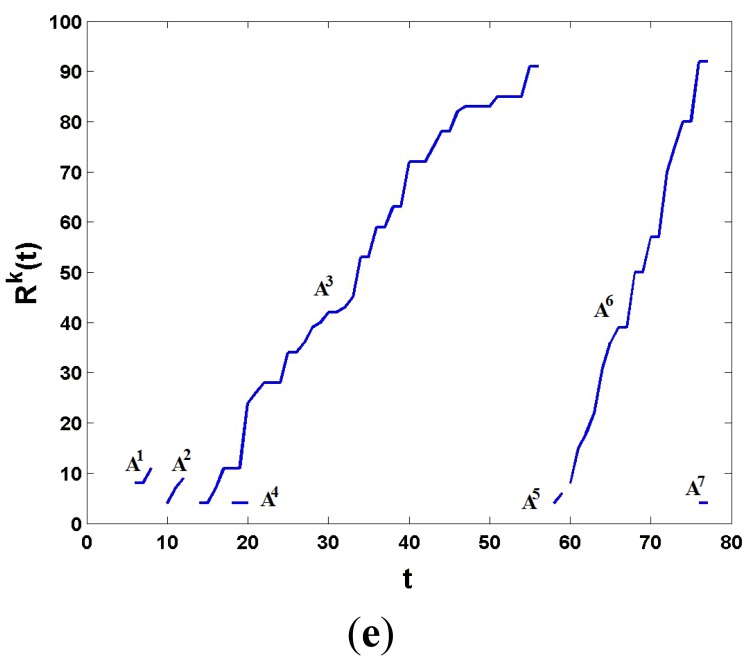
Behavior of *R^k^*(*t*), *k* = 1…*K* of a video sequences with smoke.

**Figure 10.**
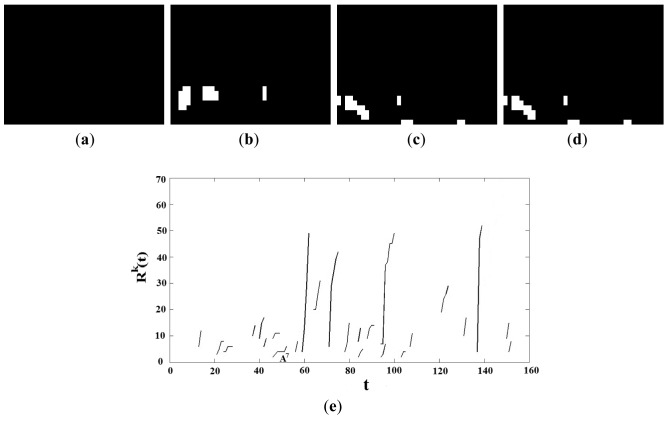
Behavior of *k* = 1…*K* of a video sequences without smoke.

Figure 9 shows the detected smoke candidate regions through the time together with the behavior of *R^k^*(*t*), *k* = 1…*K*, of the video frames with smoke, given by Figure 8(a). Figure 9(a–d) is binary images *M̂_t_* when *t* = 20, 30, 40 and 50, respectively, and Figure 9(e) shows the behavior of *R^k^*(*t*), *k* = 1…7 through time (from *t* = 0 to *t* = 80). Since the frame rate of this video is 10 frames/s, it follows that *th_5_* = 10. Taking in account the proposed condition given by Equation (22), from Figure 9(e) the candidate regions *A^1^*, *A^2^*, *A^4^*, *A^5^* and *A^7^* are discarded, because the condition is not satisfied. The proposed algorithm starts monitoring the candidate region *A^3^* during one second (*th_5_* = 10 frames) from *t* = 14, and *R*^3^(*t*) with *t* = 14 and *t* = 24 are calculated, here *R*^3^(14) is equal to 4, while *R*^3^(24) is equal to 29. Then it is clear that *R*^3^(24) > *th*_6_ ×*R*^3^(14) = 1.5 × 4 = 6. Then the proposed scheme detects the presence of smoke in *t* = 24, which corresponds to 2.4 second. Although the region *A^6^* can be considered as a smoke region, the proposed scheme detected the smoke in *t* = 24, so this region is not monitored any more. Figure 10 shows the detected smoke candidate region through the time together with the behavior of *R^k^*(*t*), *k* = 1…*K*, of the video without smoke, given by Figure 8(c), in which (a)–(d) are the binary images *M̂t*, when *t* = 20, 40, 60 and 80, respectively, and (e) shows the behavior of *R^k^*(*t*), *k* = 1…24 through time (from *t* = 0 to *t* = 80). The frame rate of this video sequence is also 10 frames/sec. In this case a lot of candidate regions are detected, however no candidate region satisfied the condition given by Equation (22), because the candidate regions with largest duration (in case of *A^7^*) is only 6 frames, which is shorter than one second, and then the proposed scheme did not detect the presence of smoke during observation time (all frames of the video).

**Figure 11.**
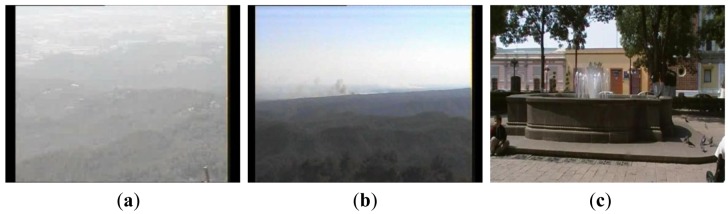
Video sequences in which the proposed scheme does not performs correctly.

The proposed smoke detection algorithm is evaluated in terms of false positive error and false negative error rates using 50 video sequences with smoke and 50 video sequences without smoke, where the proposed scheme detected correctly 48 sequences with smoke and 49 sequences without smoke. That is in two cases the proposed algorithm was not able to detect the smoke because, among other reasons, the background and smoke color is quite similar and the camera is very far from the smoke origin as shown in Figure 11(a,b). On the other hand the scheme detects smoke wrongly in one case, because the behavior of water of the fountain is quite similar to that of the smoke as shown in Figure 11(c).

The proposed system was also evaluated using the criterion proposed by [18] including correct detection (TP) and correct rejection rates (TN), false alarm and missed detection rates. Two criteria regarding the sensitivity of the wildfire observer, true positive rates (cd) and false positive rate (fd); as well as two criteria regarding the specificity, that is the true negative rate (cr) and the missed detection (md). Other evaluation measures, well known in signal detection theory and error analysis that can be also used are: the accuracy (acc), the positive predicted value (ppv) and the Mattews correlation (mmc). The evaluation results of proposed scheme, using the above mentioned criterions is shown in Table 1.

**Figure 12.**
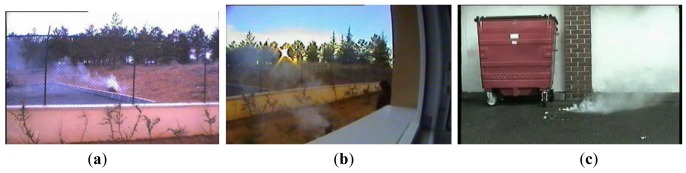
Video sequences used to obtain the evaluation results shown in Table 2. (**a**) Movie 1, (**b**) Movie 2 and (**c**) Movie 3.

Other important feature is the number of frames required to achieve a correct detection. Table 2 shows the performance of proposed method compared with two smoke detection schemes reported in the literature ([5] and [11]), using the video frames shown in Figure 12.

Evaluation results show that proposed scheme is able to detect the smoke presence faster than other previously reported algorithms [5,11].

## 4. Conclusions

In this paper we have proposed an early fire detection scheme using Internet Protocol (IP) camera technology with Motion JPEG (MJPEG) codec, in which the Discrete Cosine Transform (DCT) coefficients of each block of size 8 × 8 are available as input data. In the proposed scheme, several smoke features, such as motion, color and expansion properties are analyzed directly in the DCT domain, avoiding high time-consuming inverse DCT operations. To increase the accuracy of smoke property estimation, the DCT Inter-transformation [14,15] is introduced as a preprocessing operation, which allows changing the block size from 8 × 8 to 4 × 4 without inverse DCT. The proposed scheme is evaluated using 50 video sequences with smoke and other 50 video sequences without smoke, obtaining false positive error rates of about 2% and false negative error rates approximately equal to 4%. The principal reason for the false negative errors occurring in two video sequences with smoke is the great similarity of color between the background and smoke; this problem may be solved using other IP cameras located in other positions. The smoke detection speed of the proposed algorithm is set within one second by the threshold *th_5_* in Equation (22), as a warranty of early fire detection, which is faster than other, previously reported algorithms [5,11]. The proposed algorithm can be implemented in IP camera networks, where each IP camera can transmit its analysis results to a C4 operation center to obtain more reliable information about the fire, such as the origin, magnitude, growth speed and orientation, *etc.*

## Figures and Tables

**Table 1.** Evaluation results of proposed algorithm using global evaluation measures.

Criterion	TP	TN	FP	FN	cd	fd	cr	md	acc	PPV	mcc
	48	49	1	2	0.96	0.02	0.98	0.04	0.97	0.98	0.94

**Table 2.** Smoke detection performance comparison of proposed and two previously reported methods.

**Video Sequences**	**Duration (frames)**	**Proposed method**	**Chunyu's *et al.* method**	**Tereyin's method**	**Video descriptions**

Movie 1	630	68 frames	118 frames	132 frames	Smoke behind the fence
Movie 2	240	105 frames	121 frames	127 frames	Smoke behind window
Movie 3	900	38 frames	86 frames	98 frames	Smoke behind waste basket
